# The $\overline{B}$ → *π* form factors from QCD and their impact on *|V*_*ub*_*|*

**DOI:** 10.1007/JHEP07(2021)036

**Published:** 2021-07-08

**Authors:** Domagoj Leljak, Blaženka Melić, Danny van Dyk

**Affiliations:** 1 Rudjer Boskovic Institute, Division of Theoretical Physics, Bijenička 54, HR-10000 Zagreb, Croatia; 2 Technische Universität München, James-Franck-Straße 1, 85748 Garching, Germany

**Keywords:** QCD Phenomenology

## Abstract

We revisit light-cone sum rules with pion distribution amplitudes to determine the full set of local $\overline{B}$ → *π* form factors. To this end, we determine all duality threshold parameters from a Bayesian fit for the first time. Our results, obtained at small momentum transfer *q*^2^, are extrapolated to large *q*^2^ where they agree with precise lattice QCD results. We find that a modification to the commonly used BCL parametrization is crucial to interpolate the scalar form factor between the two *q*^2^ regions. We provide numerical results for the form factor parameters — including their covariance — based on simultaneous fit of all three form factors to both the sum rule and lattice QCD results. Our predictions for the form factors agree well with measurements of the *q*^2^ spectrum of the semileptonic decay ${\overline{B}}^0\to {\pi}^{+}{\mathrm{\ell}}^{-}{\overline{\nu}}_{\mathrm{\ell}}$. From the world average of the latter we obtain |*V*_*ub*_| = (3*.*77 ± 0*.*15) · 10^*−*3^, which is in agreement with the most recent inclusive determination at the 1 *σ* level.

**Supplementary Information:**

The online version contains supplementary material available at 10.1007/JHEP07(2021)036.

## Supplementary Information

ESM 1 (TGZ 3 kb)
